# One Health, Two Species: Linking Domestication to Cognitive Aging in Dogs and Humans

**DOI:** 10.3390/ani15192851

**Published:** 2025-09-29

**Authors:** Corinne Quadalti

**Affiliations:** 1 Department of Pharmacy and Biotechnology (FABIT), University of Bologna, 40126 Bologna, Italy; corinne.quadalti2@unibo.it; 2 Interdepartmental Centre for Industrial Research in Health Sciences and Technology ICIR HST, University of Bologna, 40126 Bologna, Italy

**Keywords:** domestication, canine cognitive dysfunction, Alzheimer’s disease, One Health

## Abstract

This commentary explores the parallel neuroanatomical and neurobiological evolution that ultimately led to modern dogs and humans, through domestication and self-domestication, respectively. The selective pressures for benignness and enhanced prosociality have reshaped brain anatomy and cognitive architecture in both *Canis familiaris* and *Homo sapiens*, leading to analogous changes including a reduction in limbic system volume and expansion of the prefrontal cortex, critical for executive control and social cognition. From a molecular point of view, shared genetic and epigenetic underpinnings of these adaptations and their implications gave rise to parallel trajectories in brain aging; notably, the emergence of canine cognitive dysfunction. Interestingly, this canine age-related cognitive decline presents significant overlaps with Alzheimer’s disease in terms of both behavioral presentation and underlying pathology. In the context of a One Health perspective, the profound influence of shared environmental exposures, such as urbanization, pollutants, and stressors, on neurodevelopment, cognitive aging, and disease susceptibility offers a compelling translational model for understanding brain health within intertwined ecological and social contexts.

## Simple Summary

Domestication has influenced not only the behavior and morphology of animals, but also their neural and cognitive architecture. While the domestic dog (*Canis familiaris*) represents a canonical example of human-directed domestication, emerging theories suggest that humans themselves may have undergone a process of self-domestication. This paper examines the neuroanatomical and cognitive aspects of modern dogs and humans, with a focus on brain structure, aging, and behavioral traits. Drawing from recent neuroimaging, genetic, and comparative anatomical studies, we comment on the parallel evolutionary trajectories followed by the two species, with particular focus on the brain structure, which underwent convergent changes in dogs and humans, including reductions in subcortical structures linked to aggressiveness and expansions of cortical regions supporting social cognition and executive functions. We further examine the One Health implications of this parallel evolution, emphasizing the role of shared environments and sociocultural niches in synchronizing aspects of neural plasticity, stress resilience, and aging in dogs and humans. The paper further focuses on the striking parallels in cognitive aging trajectories, highlighting common patterns of memory decline, prefrontal vulnerability, social selectivity, and Alzheimer’s-like pathology.

## 1. The Evolutionary Perspective

The domestication process, understood as the set of adaptations and genetic changes caused by living in a human-dominated environment, together with the active human-made selective breeding of organisms for traits beneficial to human societies, has not only reshaped the morphology and behavior of various species but also exerted profound influences on their brain anatomy and cognitive architecture [1,2,3]. Among domesticated animals, the domestic dog (*Canis familiaris*) stands out as a model species for understanding how selective pressures toward benignness and social interaction have influenced neural and behavioral evolution [4]. Concurrently, recent developments in evolutionary anthropology suggested that humans themselves (*Homo sapiens*) may have undergone a process of self-domestication, whereby our own species evolved to reduce reactive intragroup aggression and enhance prosocial behaviors, ultimately shaping both brain structure and cognitive capacities [5,6]. In an evolutionary perspective (Table 1), more than two million years ago, the rise in the genus *Homo* (i.e., *H. habilis*, *H. erectus*) saw the development of larger brains, the capacity for tool-making and use, and the onset of social collaboration. From an anatomical point of view, modern humans were originally found in Africa, where *Homo sapiens* left evidence of symbolic behavior, empathy, prosociality, and possible early signs of self-domestication like reduced skeletal robusticity and decreased reactive aggression [7]. The first evidence of potential proto-domestication of wolves in human campsites (mutualism) appeared along with the expansion of human language, art, and burial practices. In particular, genetic divergence between dogs (*Canis familiaris*) and wolves (*Canis lupus*) was dated back to 36,000–15,000 years ago. Thus, dogs were the earliest domesticated animals, differing with other species from a temporal and a geographical point of view [8,9]. For the purpose of this *Commentary*, aspects linked uniquely to the human/dog parallel evolution will be deepened.

From this moment on, evidence of parallel evolution-related pressures involving humans and dogs increased in the form of shared diets, microbiota, and disease exposures. Moreover, with the rise in early civilization (i.e., Mesopotamia, Egypt, Indus Valley), new environmental pressures occurred (i.e., pollution, zoonoses) on both species. The advent of “specialized” dogs can be dated as far as 16,000 years ago, when specialized dog breeding was performed to generate improved dogs for specific tasks (i.e., hunting, guarding) [10], and later the Romans and the early Chinese dynasties generated dogs with companion roles that became prominent in elite societies [11,12]. These selective processes sparked the diversification of morphotypes and neuroanatomical variation that we observe, leading up to the present day, with the industrial and scientific age being characterized by the rise in eugenics and formal breed standards [13]. Finally, the 20th and 21st centuries are defined as “the genomic era”, where thanks to novel technologies applied to DNA, multiple domestication events were confirmed in dogs, and epigenetic studies underlined the fundamental role of early-life environment in the determination of heritable changes. Importantly, the rapidly and constantly increasing process of urbanization led to the concentration of domestic dogs and humans in dense, shared life environments, with the parallel appearance of shared aging-related pathologies (i.e., cognitive dysfunction, neurodegeneration), reinforcing attention to environmental stress, diet, and pollution as shared brain health determinants [14,15].

## 2. Domestication and Neural Evolution

Domestication is traditionally associated with morphological and behavioral changes, commonly referred to as the domestication syndrome [16]. A hallmark of domestication across mammals is the reduction in relative brain size, especially in regions linked to fear and aggression [17]. Fossil records indicate that *Homo sapiens* experienced a 10% to 15% reduction in brain volume over the past 30,000 years, particularly in the limbic system [18]. Meanwhile, the prefrontal cortex, associated with executive control, social inhibition, and planning, underwent relative expansion, a series of structural brain changes indicating the evolution of humans toward prosociality, including empathy, alliance formation, and language [18,19]. This documented historical evolution of human brain morphology and behavior is at the basis of the self-domestication hypothesis, explaining the anatomical (cranial) and behavioral changes as the result of social selection against intra-group aggression and in favor of social cooperation [20,21]. The self-domestication-related changes allowed for improved conflict resolution, alliance formation, and the emergence of complex language and culture. Modern humans, comparably to modern dogs, appear to have evolved a “friendliness niche,” favoring tolerance and behavioral plasticity [22].

The domestication syndrome in dogs includes the abovementioned brain reduction, of >24% [23] and distinctive morphological traits (i.e., floppy ears, reduced snout length) coupled with neurodevelopmental traits (i.e., benignness, increased sociability), the latter thought to arise from neural crest cell modifications [24]. A neural crest domestication syndrome (NCDS) hypothesis has been postulated, suggesting that domestication-related selective pressures for the above-cited behavioral traits carried over a decreased neural crest cell proliferation and migration, consequently affecting the cranio-facial anatomy of domesticated individuals and thus providing a mechanistic explanation for a whole bunch of common traits observed among domesticated species. A validation of the NCDS hypothesis was recently provided by a study focusing on the divergent development of larynges, a neural-crest derived organ, in wolves and domestic dogs, confirming that the latter had absolutely and relatively shorter vocal folds and smaller larynges compared to the ancestral counterpart [25].

Interestingly, likewise to what is described in humans, the selection for benignness, communication, and visual/social processing, together with behavioral traits like attachment, gaze-following, and sensitivity to human emotions, was coupled also by a reduction in amygdalar volume and an expansion of the neocortex as described by MRI and morphological studies in modern dogs [26].

### 2.1. Impact of Domestication on Brain Anatomy

Though different in size and complexity, the human and the canine brains show functional and structural homologies, particularly in regions affected by domestication and aging.

The adult human brain is approximately 1200–1400 g, with an encephalization quotient (EQ) ranging from 7.4 to 7.8, indicating a high brain-to-body size ratio and a substantial neocortex-to-brain ratio [27]. In contrast, dog brain size varies widely by breed (i.e., ~60–150 g), and EQ of dogs is about 1.2, which is higher than that of many non-primate mammals, in particular among working and companion breeds [28,29]. Despite size differences, the basic architectural layout of the dog brain remains conserved, with clearly delineated telencephalon, diencephalon, mesencephalon, metencephalon, and myelencephalon [30].

The neocortex represents ~80% of human brain volume and is highly elaborated, supporting language, abstract reasoning, and self-reflection. Conversely, the neocortex constitutes only ~30% of a dog’s brain volume, but, most importantly, it retains functional areas related to vision, somatosensation, and motor planning. Recent comparative functional MRI studies showed that dogs have active cortical responses to social stimuli like human faces, vocal tones, and even language-like cues [31], suggesting a socially attuned neocortical organization in dogs, even if the degree of organization is comparatively lower than that observed in humans.

Among cortical areas, the prefrontal cortex (PFC) supports executive functions such as impulse control, decision-making, and empathy. In humans, the PFC is disproportionately large and intricately connected. Dogs also possess a PFC, but it is relatively smaller and more rudimentary in cortical layering and association tracts. However, canine PFC activity correlates with behaviors like delayed gratification, training success, and problem-solving, implying evolutionary conservation of function with species-specific elaboration [32].

The limbic system governs emotion, fear responses, and memory. The amygdala, hippocampus, and septum are present in both humans and dogs. Domesticated dogs show reduced amygdala volume and reactivity compared to wolves, consistent with decreased aggression and increased social tolerance [26]. Similarly, human self-domestication is associated with reduced limbic volume and enhanced top-down regulation via the PFC.

The hippocampus is a key area for spatial navigation and episodic memory, and it is structurally conserved across mammals. In humans, the hippocampus is heavily integrated with the visual cortex, being vision the primary sense. On the other hand, dogs retain exceptional olfactory brain structures and networks, including an enlarged olfactory bulb and accessory olfactory system, while humans have a relatively vestigial olfactory lobe. By contrast, humans possess more cortical elaboration in the auditory-associated areas. Interestingly, dogs show specialized regions for processing human vocalizations and emotional prosody [33]. Notably, dogs rely heavily on hippocampal circuits for territory mapping, social memory, and face recognition [34].

Several imaging studies have explored the brain networks’ activation patterns, using paradigms to explore dog-human interaction, overall revealing conserved default-mode networks (DMN)-like patterns in dogs, particularly involving the medial PFC and posterior parietal areas. Humans exhibit a more complex and robust DMN, yet the identification of similar core nodes in dogs supports a shared substrate for introspective and social-cognitive processing [35].

Differences in white matter structure and organization could be part of these different functional patterns. Human white matter tracts show high plasticity and are crucial for language and consciousness, while the corpus callosum in dogs is smaller in absolute and relative size, reflecting fewer interhemispheric integrations [36].

It is important to mention that dog breeds display striking neuroanatomical variation, especially in cortex folding, skull shape (i.e., brachycephalic vs. dolichocephalic), and cerebellar architecture [26]. The risk of potential breed-related bias must be considered when exploring any kind of comparative results (i.e., intra-specific, between domestic dogs and wolves, or inter-specific, between dogs and humans).

### 2.2. Genetic and Epigenetic Underpinnings of Domestication and Brain Evolution

While evolutionarily related behavioral and neuroanatomical changes are well documented, the genetic mechanisms underlying these transformations remain an active field of inquiry. Increasing attention was turned not only to the static genome, but also to the epigenetic regulation patterns as a dynamic interface between environmental input and inherited neural traits. The parallel evolution of humans and dogs appears to have shaped the expression of genes involved in neural crest development, neurotransmission, and social cognition [37,38].

Since the availability of the whole dog genome [39], several genes associated with domestication-related traits have been identified across species. In dogs, selection has acted on genes linked to behavior, such as SEZ6L, GTF2I, and OXTR, which modulate social bonding, fear response, and synaptic plasticity [40].

Canine domestication has exerted a profound and diverse selective pressure, extending beyond behavioral aspects to significantly impact genetic selection and, consequently, morphology. A striking example of this influence is the extraordinary variability in body size observed in dog breeds, which is the widest among all terrestrial vertebrates [41]. This diversity, which can span a 40-fold difference between the smallest and largest breeds, primarily stems from intense selection, in particular over the last two centuries, as breeders propagated extreme phenotypes [42]. Among approximately 20 genes identified as regulating canine body size, the insulin-like growth factor 1 (IGF-1) gene plays a predominant role, controlling about 15% of the body size variation among breeds [42]. A single variant predominates in modern gray wolves and large domestic breeds, while the ancestral allele, which predisposes to small size, was common in small-sized breeds and smaller wild canids. Analyses indicate that this ancestral allele, a key regulator of canid body size, had nearly vanished in Pleistocene wolves before its recent resurgence due to human-imposed selection for small-sized dog breeds [41,42].

Comparative genomics showed overlap between domesticated dogs and humans in genes influencing neural crest cell migration [24]. These genes regulate craniofacial morphology and brain structure, specifically in areas modulating emotion and aggression. Recent advances in transcriptomics have revealed domestication-associated shifts in gene expression profiles in the prefrontal cortex and limbic structures by comparing domesticated and wild animals. These shifts include genes modulating neural excitability, neuroinflammation, and cortical layering, contributing to the behavioral phenotypes typical of domesticated species [43,44,45].

Beyond DNA sequence, epigenetic modifications such as DNA methylation, histone acetylation, and non-coding RNAs contribute to gene regulation in response to environmental cues. In both humans and dogs, early-life stress, nutrition, toxin exposure, and social environment influence the epigenetic landscape of the brain. In dogs, studies have shown that methylation patterns in genes related to hypothalamic–pituitary–adrenal (HPA) axis function correlate with fearfulness and social anxiety [46]. Analogously, in humans, adverse childhood experiences leave enduring epigenetic marks on genes governing cortisol sensitivity and neuroimmune function [47].

Such modifications can persist across generations, suggesting that domestication may have involved not just selection on gene variants, but also heritable epigenetic programming [48], accelerating cognitive and emotional evolution by selecting for individuals with favorable neuroepigenetic profiles [49]. Epigenetic inheritance provides a mechanism for rapid behavioral adaptation across generations, supplementing slower genetic change.

## 3. Brain Aging and Neurodegenerative Diseases

### 3.1. Brain Aging

Anatomically, domestication and aging similarly affect the same brain areas (i.e., amygdala, prefrontal cortex, hippocampus) in both humans and dogs, causing structural and functional changes. Subcortical structures, especially the amygdala and hypothalamus, shrink or reorganize following both domestication and age. In contrast, domestication is associated with the expansion of neocortical and associative areas, which are also targets for degenerative age-related processes [50,51]. On the one hand, domestication promotes social-emotional plasticity; while on the other, aging progressively narrows cognitive flexibility [52]. Consequently, the cognitive domains that are enhanced during evolution, such as social cognition, spatial orientation, and emotional regulation, become particularly vulnerable during senescence [53,54]. This creates a life-history arc that ties together domestication and cognitive aging, in both physiological and pathological conditions [14].

The parallel trajectory suggests the forces that optimized social behavior and cooperation in both species make similar domains sensitive to decline. In fact, social selectivity and preference for routine are common features of aging in both species, suggesting shared socioemotional aging mechanisms [55].

In domestic dogs, aging is often associated with a spectrum of cognitive and neuroanatomical changes known as Canine cognitive dysfunction (CCD) [56]. CCD is a chronic and progressive neurodegenerative condition that can range from mild cognitive impairments to severe cognitive alterations that compromise quality of life [57,58].

The prevalence of CCD increases with age, affecting 28% of dogs aged 11–12 years and up to 68% of dogs aged 15–16 years. The progression of the disease is gradual and can be classified into stages similar to human Alzheimer’s disease [59]. The diagnosis of CCD relies on the exclusion of other pathologies with similar clinical presentation (i.e., brain tumors, metabolic imbalances, etc.), with the major challenge being the near-total reliance on owner observations, which can be highly subjective and influenced by their perceptions. Moreover, the initial behavioral changes are often subtle, leading to late diagnosis [58,59].

Due to the relatively short lifespan, dogs represent a spontaneous and accelerated model of aging of invaluable importance for longitudinal and comparative studies of human age-related cognitive dysfunctions [57].

### 3.2. Alzheimer’s and Alzheimer’s-like Symptoms and Neuropathology

Canine cognitive dysfunction (CCD) is increasingly recognized as a naturally occurring model of human Alzheimer’s disease (AD), with significant overlap in both clinical presentation and underlying pathology [59,60,61,62,63]. The clinical signs of CCD are summarized by the acronym DISHAA: disorientation, altered social interactions, altered sleep–wake cycles, house soiling, altered activity levels, and increased anxiety [58,59]. These symptoms closely mirror those of human AD, which include memory impairment, executive dysfunction, disorientation, and emotional disturbances. The similarities between CCD and AD extend to the neuropathological level. Both CCD-affected dogs and AD patients present with cortical atrophy, impacting mainly the hippocampus and prefrontal cortex [61,64]. Brain atrophy in dogs can be confirmed by Magnetic Resonance Imaging, and when combined with clinical signs, is diagnostic for CCD [58,59]. CCD is characterized by the extracellular accumulation of β-amyloid plaques in prefrontal and temporal lobes [58,59], comparable to the early stages of the pathological cascade of human AD [65]. Other similarities between CCD dogs and AD patients can be found in increased phosphorylated tau protein, oxidative stress, neuroinflammation, and cholinergic deficits [58,59]. Importantly, a notable difference is that one of the hallmarks of AD, the neurofibrillary tangles of tau protein, is rare or absent in dogs [62], suggesting an incomplete model for AD, but still one of high translational value. This parallel is further supported by evidence in CCD-affected dogs of progressive cortical thinning and increased ventricular volume revealed by MRI studies, compromised neuronal metabolism as indicated by increased markers of oxidative damage, and reduced expression of acetylcholine and related enzymes. These findings, and the responsiveness of CCD-affected dogs to cholinesterase inhibitors and dietary antioxidants in early trials [66], highlight the common nature of cognitive decline in dogs and humans [61].

Similarities extend also to a molecular level. For instance, P-glycoprotein is an efflux pump involved in β-amyloid transport, and its decreased function is linked to β-amyloid accumulation in AD. Some dog breeds, like the “ivermectin-sensitive” Collies, have a genetic deficiency of P-glycoprotein, offering a natural model to study this transporter’s role in neurodegeneration [58].

These parallels provide a robust translational framework. Different initiatives are working in parallel to exploit the potential of dogs as natural models of human aging, as well as for developing a parallel therapeutic strategy under the One Health approach. In one study, a canine model was exploited for the development of a preclinical platform based on trained beagles to unveil correlations between aging, neuropathology, and cognitive profile [57]. In parallel, the DOGMA (Dogs Overcoming Geriatric Memory and Aging) Initiative was created, embodying the One Health approach. DOGMA’s research goals include correlating biomarker levels with biometric data from smart collars, mapping the longitudinal progression of the disease, and creating a global database to establish validated reference values for early diagnosis. This type of research, conducted in real-world veterinary clinical settings, will not only improve the diagnosis and treatment of CCD but will also provide crucial comparative data for developing therapies for human dementia [58,59].

## 4. Parallel Evolution in Shared Environments: A One Health and Translational Perspective

The long-term cohabitation of domestic dogs and humans represents a unique case of co-exposure to shared environmental and social contexts, a core tenet of the One Health framework [67]. Both species evolved not only in close physical proximity but also under similar ecological, emotional, and behavioral pressures. Domestic dogs adapted to human-built environments, food sources, rhythms of daily life, and emotional signals, while humans benefited from enhanced security, cooperation, and companionship.

These overlapping experiences, which has been going on for tens of thousands of years, in addition to the co-exposure to stressors and pathogens, likely promoted parallel neurobiological adaptations, particularly in brain regions associated with social bonding, sensory integration, and environmental stress response. Domestication, therefore, should not be viewed solely as a unilateral process of human-directed selection, but rather as a mutual reshaping of biology under shared ecological and social constraints.

The coexistence of dogs and humans is at the basis of the evolution of parallel response pathways for stress regulation, oxytocin signaling, and emotional attunement [68], in addition to sculpting overlapping neural architectures in favor of attachment and behavioral flexibility.

The One Health approach highlights how intertwined ecological and social environments influence brain development, aging, and disease susceptibility across species, also including environmental health. The health of individuals and species is, in fact, deeply embedded in their surrounding ecological context. The One Health framework acknowledges that human and animal health are inseparably linked to the health of the ecosystems in which they cohabit, which influence neurodevelopment, cognitive aging, and disease vulnerability.

In evolutionary terms, environmental health encompasses resource availability, pathogen load, climate stability, and exposure to toxins or stressors. For both humans and dogs, ecological pressures have shaped brain function and behavior via natural and artificial selection. In ancestral environments, survival pressure favored individuals with robust stress-response systems, efficient foraging strategies, and adaptive social cognition.

Domestication aligned the canine evolutionary path with human-altered environments characterized by settlements, agricultural zones, and eventually industrial habitats. These new landscapes selected for decreased fight response [4,69], tolerance to novelty [70], and flexibility in dietary [71] and spatial behaviors [72]. Similarly, human populations adapting to post-Pleistocene environments, characterized by urbanization, zoonoses, and crowding, also saw selection for cognitive flexibility, reduced aggression, and group-level coordination.

Environmental adversity, including exposure to poor air quality, pollutants, pathogens, and nutritional stress, exerts a shaping force on brain development, behavioral adaptation capacities, and age-related brain vulnerability. The environments shared by humans and dogs, including households, urban spaces, and natural ecosystems, can modulate neurodevelopmental trajectories, as also experimentally demonstrated in laboratory animals [73] via endocrine-disrupting chemicals (i.e., bisphenol A, pesticides) [74], chronic noise pollution [75], dietary composition and additives, microbiome alterations from antibiotics or processed food [76,77], and sedentary lifestyle [78]. Importantly, the neurobiological trajectory shaped by the shared environment is also involved in pathological brain conditions. For example, air pollution such as fine particulate matter (i.e., PM2.5) has been recently included among the so-called “modifiable risk factors for Alzheimer’s disease” [79]. Moreover, human epidemiological studies and controlled animal studies have shown that exposure to air pollution may lead to neurotoxicity [80]. Dogs exposed to Mexico City air pollution showed symptoms of chronic inflammation, neurodegeneration, and DNA damage in various brain regions, and comparable neuropathological lesions in the prefrontal cortex were observed in both children and dogs [15,81].

Environmental enrichment is known to enhance cortical thickness and synaptogenesis in laboratory animals, and similar effects are described in pet dogs provided with varied stimuli, social contact, and exercise [82]. However, impoverished environments correlate with increased cortisol levels [83], hippocampal shrinkage [84], and behavioral rigidity [85]. Environmental pathogens also play a role in neurocognitive health. Shared zoonotic risks (i.e., Toxoplasma gondii, Leptospira) can cause central nervous system infections or chronic inflammation [86,87].

The overall environmental conditions that supported dog-human parallel evolution are subject to inevitable changes related to the phenomenon known as climate change. This phenomenon introduces new stressors like heat stress, vector-borne diseases, and food insecurity. As a consequence, cognitive development, emotional regulation, and life expectancy are affected. Dogs often act as early indicators of environmental health threats, offering a sentinel species perspective within One Health surveillance.

## 5. Conclusions

The domestication of dogs and the self-domestication of humans represent remark-able case studies in convergent evolution and offer a compelling lens through which to understand not only human and dog evolution but also translational models. Despite the phylogenetic distance, both species exhibit strikingly similar patterns in brain development, cognitive aging, social behavior, and even susceptibility to neurodegenerative diseases. This convergence is not merely a product of chance but reflects shared evolutionary pressures, including selection for social tolerance, behavioral plasticity, and emotional intelligence within cohabiting ecosystems.

Comparative neuroanatomy between humans and dogs reveals structural parallels in the limbic system, prefrontal cortex, and hippocampus, regions critically involved in social cognition, learning, and memory, which are particularly sensitive to aging and environmental inputs. They also represent common targets for degenerative processes such as canine cognitive dysfunction and Alzheimer’s disease.

From a One Health perspective, the intertwined lives of dogs and humans extend beyond companionship to shared vulnerabilities to pollutants, dietary changes, emotional stressors, and pathogens. Environmental health is therefore not an external variable but an active force shaping brain plasticity, disease susceptibility, and behavioral phenotypes in both species.

A recent perspective article [88] proposed a data-collection strategy to gather human and canine sanitary data with a focus on related geocoded environmental information and other aspects of the so-called exposome (i.e., socioeconomic characteristics of the neighborhood, climate, air quality, primary nutrition sources, proximity and access to health care and veterinary facilities, etc.). The proposed data-collection approach could provide a substantial benefit to both species, giving real substance to the One Health concept.

## Figures and Tables

**Table 1:** Timeline of domestication in humans and dogs.

Era/Time Period	Human Milestones	Dog Milestones	Shared Adaptation
>2 mya	Early *Homo* evolution, tool use, social groups	No domestication	Foundation of social cognition
~100 kya	Anatomically modern humans	Wild wolves coexisting	Selection for benignness (self-domestication/handraising)
~50–30 kya	Behavioral modernity, symbolic culture, art, language	Proto-domestication; early wolf-human mutualism	Mutual social attentiveness
~20–15 kya	Early dog-human cohabitation	Dog genetic divergence from wolves	Selection for tameness, cooperation
~12–10 kya	Neolithic Revolution: agriculture	Dogs help herd, guard, scavenge	Shared diets, microbiomes, pathogens
~8–4 kya	Urban societies, early empires	Dogs in art, status symbols	Co-adaptation to urban life; urban ecological pressures
~2 kya	Classical societies, cognitive specialization	Early breed diversification (Roman/Chinese records)	Trainability, breed-specific behaviors
18th–19th c.	Scientific and Industrial Revolutions	Standardized breeding	Shared stress and emotional labor roles
20th–21st c.	Genomic era, One Health awareness, aging/neurodegeneration studies	Companion animals, genetic models, cognitive disorder research	Shared cognitive aging, environmental sensitivity, disease susceptibility

mya = million years ago; kya = kilo years ago (kilo corresponds to a thousand); c. = centuries.

## Data Availability

Not applicable.

## Abbreviations

The following abbreviations are used in this manuscript:

AD	Alzheimer’s disease
CCD	canine cognitive dysfunction
c.	centuries
DMN	default-mode networks
EQ	encephalization quotient
HPA	hypothalamic–pituitary–adrenal (axis)
NCDS	neural crest domestication syndrome
NFTs	neurofibrillary tangles
MRI	magnetic resonance imaging
Mya	million years ago
Kya	kilo years ago (kilo corresponds to a thousand)
PFC	prefrontal cortex
